# Cross-Cultural Adaptation and Quantitative Evaluation of Dysfunctional Voiding and Incontinence Scoring System in Pediatric Serbian Population

**DOI:** 10.3390/medicina55040100

**Published:** 2019-04-11

**Authors:** Dragana Cirovic, Ivana Petronic, Jasna Stojkovic, Ivan Soldatovic, Polina Pavicevic, Marta Bizic, Vesna Bokan-Mirkovic, Tatjana Knezevic, Dejan Nikolic

**Affiliations:** 1 Faculty of Medicine, Unviersity of Belgrade, 11000 Belgrade, Serbia; cirovicdragana@yahoo.com (D.C.); ivana.pm@live.com (I.P.); stojkovic.jasna@yahoo.com (J.S.); soldatovic.ivan@gmail.com (I.S.); pzmbov@yahoo.com (P.P.); martabizic@gmail.com (M.B.); 2 Department of Physical Medicine and Rehabilitation, University Childrens Hospital, 11000 Belgrade, Serbia; tknezevic@gmail.com; 3 Department of Radiology, University Childrens Hospital, 11000 Belgrade, Serbia; 4 Department of Pediatric Surgery, University Childrens Hospital, 11000 Belgrade, Serbia; 5 Clinical Centre of Montenegro, 81000 Podgorica, Montenegro; vesnabokanmir@gmail.com

**Keywords:** dysfunctional voiding, incontinence, validation, questionnaire, children

## Abstract

*Background and objective*: Dysfunctional voiding (DV) presents relatively frequent problem in pediatric urologist practice. The necessity for implementation of DV evaluation in the pediatric population is of particular importance, since there is no clear consensus on the clinical assessment of such condition. The aims of our study were to evaluate the test/retest reliability and reproducibility of dysfunctional voiding and incontinence scoring system: Serbian version (DVISS_SR_) in patients with voiding and incontinence dysfunctions without structural deformities, and to estimate cut-off value for DVISS_SR_. *Methods:* The cross-sectional study included 57 children with voiding and incontinence dysfunctions and 30 healthy pediatric controls. For the evaluation of voiding and incontinence dysfunction we used DVISS. The forward–backward method was applied for translation of the DVISS questionnaire from English into Serbian language. Reproducibility was analyzed by Interclass Correlation Coefficient (ICC). Sensitivity and specificity of DVISS_SR_ scores was done by receiver operating curve (ROC) curve. *Results:* There was a significant difference in DVISS_SR_ score between patients and controls (*p* < 0.001). For reliability and reproducibility of the questionnaire, there was no significant difference between repeated measurements (*p* = 0.141), and strong reliability (ICC = 0.957; *p* < 0.001). *Conclusion:* We have demonstrated successful translation and validation of the DVISS_SR_ score. Moreover, a reliable scoring system of children with voiding dysfunctions should include evaluations of symptom scoring systems at the multicentric level.

## 1. Introduction

Dysfunctional voiding (DV) presents relatively frequent problem (around 40%) in pediatric urologist practice [1]. However, Altan et al. reported that pediatric DV and urinary incontinence encompass around 10% with different severity degrees [2]. Bearing in mind that there are few studies dealing with symptom scoring in pediatric patients with functional voiding problems [3], there is a need for further studies, particularly in the domain of implementation of a scoring system. Furthermore, it was stated that there is not a generally accepted method for the quantitative evaluation of clinical symptoms in patients with DV [3].

So far, several questionnaires were proposed in the evaluation of DV in the pediatric population. In Serbia, the dysfunctional voiding symptom score (DVSS_SR_) has been validated and successfully implemented in practice. However, it was stated that the dysfunctional voiding and incontinence scoring system (DVISS) has the highest accuracy compared to DVSS and the Incontinence Symptom Index-Pediatric (ISI-P) [3].

Therefore, the aim of our study was to evaluate the test/retest reliability and reproducibility of the DVISS_SR_ in patients with voiding and incontinence dysfunctions without structural deformities. The second aim was to estimate cut-off value for the DVISS_SR_.

## 2. Material and Methods

### 2.1. Study Group

The cross-sectional study included 57 children with voiding and incontinence dysfunctions (VDI) and 30 healthy pediatric controls between 2014–2018. The diagnosis of voiding and incontinence dysfunction was done by a board certified urologist and physiatrist. Patients with structural abnormalities were excluded from the study. Regarding gender, children were grouped into males and females, while considering educational level, they were grouped into a preschool group and an elementary school group. Prior to inclusion into the study, all participants and/or parents/legal guardians were informed and consent was obtained. The study was approved by the Institutional Review Board of University Children’s Hospital (date: 28 November 2013, Nr. 26/257) and followed the principles of good clinical practice.

For the evaluation of voiding and incontinence dysfunction, we used the Dysfunctional Voiding and Incontinence Scoring System (DVISS) that was administered to the patient group twice (initially and three days after), while it was administered once (initially) to the control group. Parents or legal guardians were asked to complete the DVISS questionnaire. All parents were from different educational backgrounds, and completely collaborated without misunderstanding of the questions that were asked.

### 2.2. Methodological Instrument

The DVISS questionnaire is composed of 13 questions concerning voiding dysfunction and incontinence and one question regarding quality of life (Supplementary Material Table S1). The score range varied between 0 and 35 [4]. The first and the third questions were graded: 0, 1, 3 and 5; the second question was graded: 1, 3 and 5; the fourth question was graded: 1 and 4; the fifth, the seventh, the tenth and the thirteenth questions were graded: 0 and 1; the sixth was graded: 0 and 4; the eight, the ninth, the eleventh and the twelfth were graded: 0 and 2; while the fourteenth question (quality of life) was graded 0, 1, 2 and 3. The fourteenth question, even though it was translated, was not included in our study since it does not affect the score values.

### 2.3. Translation Process

We translated the DVISS questionnaire from English to Serbian and culturally adopted the items of the questionnaire with regards to the possible cultural differences among the different populations.

The forward–backward method was applied for translation of the DVISS questionnaire from English into Serbian [5,6]. The translation process followed the principles of framework for translation and cultural adaptation regarding patient-reported outcome measures [7]. Two authors who participated in study aim design were engaged in the translation process of the DVISS questionnaire into Serbian. In a process of forward translation, two questionnaire versions were created (DVISS I and DVISS II). In a reconciliation step, generated versions were compared, analyzed, and merged into a final forward translation (DVISS 1.0) supervised by an independent translator who was not familiar with the study objectives. The translator who participated in the translation process lived more than a year in an English-speaking country and is fluent in English and Serbian. The next step was back-translation into English by another independent translator who was fluent in English and Serbian and as well non-familiar with study objectives. The gained discrepancies between the forward and the backward translations were debated and resolved by consensus with no major linguistic discordant. The final version of the DVISS (DVISS 2.0) questionnaire (pretest) was presented probationary to the parents of nine children that were not from the study cohort for the analysis of question-item appropriateness and linguistic understanding. Parents or legal guardians were asked to arrange feedback where questions were not well understood. There was no feedback regarding difficulties in item interpretation and answering during the debriefing step. The expert panel that consisted of two independent pediatric urologists and one independent physiatrist with subspecialist interests in voiding dysfunctions [8] was invited for the final debate and the Serbian version of DVISS 3.0 was approved as the final form (DVISS_SR_).

### 2.4. Statistical Analysis

Descriptive statistics are presented using count (%), mean ± standard deviation and median (interquartile range (IQR)). Group differences are analyzed using t-tests, Mann–Whitney U test, Pearson chi square test and Fisher’s exact test, depending on data type and distribution. Normality of distribution was assessed using descriptive parameters (skewness and kurtosis), normality distribution tests (Kolmogorov–Smirnov and Shapiro–Wilk) and graphical methods (histogram, boxplot and quantile-quantile QQ plot). Within group differences are analyzed using the Wilcoxon signed rank test. Reproducibility is analyzed using the interclass correlation coefficient (ICC). Receiver operating characteristics are used to obtain the adequate cut-off value of the score (best combination of sensitivity and specificity) for the Serbian population. All *p*-values less than 0.05 were considered significant. All data were analyzed using IBM SPSS 20.0 statistical software (IBM Corporation, Armonk, NY, USA).

## 3. Results

In Table 1, we present the basic characteristics between the tested groups. There were no significant differences for age, gender and education, implying homogeneous samples. There was a significant difference in DVISS_SR_ score between patients and controls. Regarding reproducibility of the questionnaire, there is no significant difference between repeated measurements (Wilcoxon signed rank test *p* = 0.141). High reproducibility was obtained overall for the DVISS_SR_ score (ICC = 0.957; *p* < 0.001).

For the DVISS_SR_ questionnaire, there was excellent reproducibility for items 1, 2, 8, 9, 11 and 13. There was good reproducibility for items 3, 4, 5, 6, 7, 10 and 12 (Table 2).

**Figure 1:**
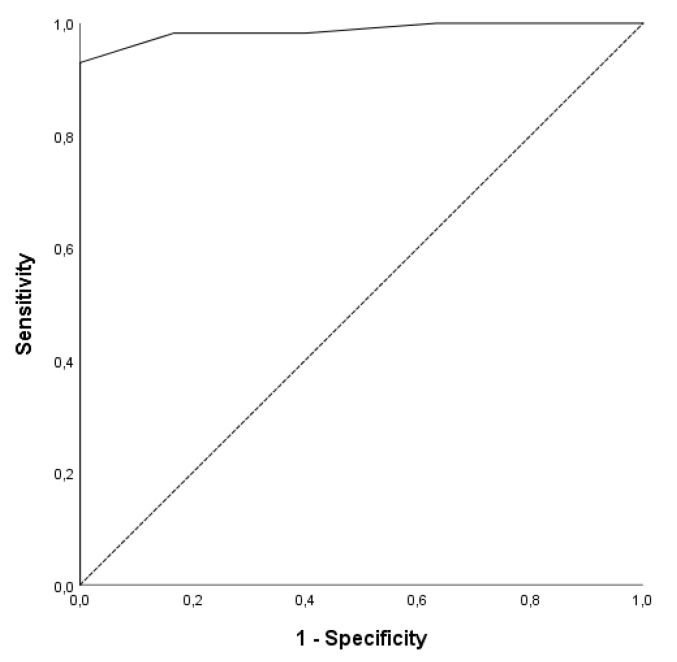
Receiver operating curve (ROC) for the DVISS_SR_ score.

The sensitivity and specificity of DVISS_SR_ score was reached by receiver operating curve (ROC). The area under the curve (AUC) is 0.987 (0.967–1.000), with significant difference (*p* < 0.001) (Figure 1).

The diagnostic characteristics of DVISS_SR_ cut-off values that were recommended and the one with best ratio of sensitivity and specificity as determined by the Youden index is presented in Table 3. There is higher sensitivity with lower DVISS_SR_ cut-off scores, while the specificity is the same.

## 4. Discussion

The necessity for implementing a voiding dysfunction evaluation in the pediatric population is of particular importance, since there is no clear consensus on the clinical assessment of such conditions. In a multicenter controlled trial, there was suggestion that urodynamic studies should be reserved for individuals who fail the standard treatment of voiding dysfunction [9].

It is known that inadequate translation of measuring instruments may result in changes in the sensitivity and specificity of original instruments, thus limiting the comparability of responses across different populations [6]. In our study, since there was not any feedback about the DVISS 2.0 version pretest, we have demonstrated successful cultural adaptation and linguistic translation of the original DVISS questionnaire into the Serbian language (DVISS_SR_) on the Serbian population.

For the purpose of obtaining the most reliable comparisons between patients and the control group, we have matched both study samples to be of similar age, gender and educational distribution.

We have demonstrated high reliability of the translated DVISS_SR_ and a higher degree of reproducibility among items. Our findings of reliability for the DVISS_SR_ were higher than in the study of Kaya Narter et al. [10]. A possible explanation for such a discrepancy could be in the different cultural and social environments.

When separate analyses for each item were performed, all were in the range of good or excellent reliability [11]. Only item 5 demonstrated the lowest but good reliability. A possible explanation for a slightly lower level of reliability for item 5 might be in local cultural beliefs of “a shame” for having frequent voiding activities in one day.

In our study, we have noticed that a higher sensitivity for the translated DVISS_SR_ was higher for lower scores (score of four vs. score of nine) compared to the previous study, where the proposed cut-off value was 8.5 with a sensitivity of 90% and a specificity of 90% [3]. Additionally, in another study, the cut-off value was proposed to be nine, with a sensitivity of 100% and a specificity of 79.4% [4]. However, our specificity did not differ between scores of four and nine, indicating the same level of power to predict those subjects without dysfunctional voiding. A lower score of DVISS_SR_ with higher sensitivity might be explained by the fact that different psychosocial and cultural patterns exist among different populations, where various degrees of parental reactions and actions are present. Namely, in the Serbian population, the increased attention to detail and even slight changes in voiding habits are concerning signs for parents to seek medical attention.

The limitation of this study refers to the study sample, where further investigation on a larger group of participants is advised for increased reliability of the tested questionnaire.

## 5. Conclusions

We have demonstrated successful translation and validation of a Serbian version of the DVISS_SR_ score. Moreover, a reliable scoring system of children with voiding dysfunctions should include evaluations of symptom scoring systems at the multicentric level.

## Figures and Tables

**Table 1:** Basic characteristics and Dysfunctional Voiding and Incontinence Scoring System: Serbian Version (DVISS_SR_) score.

	Groups		*p* Value
	DVI Group (*n* = 57)	Control Group (*n* = 30)	
Basic characteristics			
Age (years)	7.1 ± 1.5	7.1 ± 1.3	0.927 ^a^
Gender—female	29 (50.1%)	15 (50%)	0.938 ^b^
Education—elementary school	31 (54.4%)	19 (63.3%)	0.422 ^b^
DVISS_SR_ score			
Score 9+	47 (82.5%)	0	<0.001 ^c^
Score median (IQR)	17 (12)	1 (2)	<0.001 ^d^
Score (repeated) median (IQR)	17 (11)		

Results are presented as count (%), mean ± standard deviation or median (IQR). IQR – interquartile range; ^a^ t-test; ^b^ Pearson chi square test; ^c^ Fisher’s exact test; ^d^ Mann–Whitney U test.

**Table 2:** Test/retest reproducibility analysis of the Serbian version of the Dysfuncional Voiding and Incontinence Scoring System (DVISS_SR_).

Test/Retest Items	ICC
Item 1	0.907
Item 2	0.931
Item 3	0.860
Item 4	0.886
Item 5	0.797
Item 6	0.867
Item 7	0.889
Item 8	0.965
Item 9	0.929
Item 10	0.897
Item 11	0.926
Item 12	0.889
Item 13	0.906

ICC—Interclass Correlation Coefficient.

**Table 3:** Diagnostic characteristics of DVISS_SR_ score cut-off values.

Cut-Off	Sensitivity	Specificity	PPV	NPV	LR+	LR-
9+	0.82	1	1	0.75	Inf.	0.175
4+	0.93	1	1	0.88	Inf.	0.070

PPV—positive predictive value; NPV—negative predictive value; LR+—likelihood ratio of a positive test result; LR-—likelihood ratio of a negative test result; Inf.—Infinity.

## Supplementary Materials

The following are available online at https://www.mdpi.com/1010-660X/55/4/100/s1, Table S1: Serbian version of DVISS (DVISSSR).
