# Supplementary material for: Cross-Cultural Adaptation and Quantitative Evaluation of Dysfunctional Voiding and Incontinence Scoring System in Pediatric Serbian Population
Source: Medicina (Kaunas). 2019 Apr 11;55(4):100. doi: 10.3390/medicina55040100 (PMC6524058; doi:10.3390/medicina55040100)
Supplement: Supplementary file 1 [file medicina-55-00100-s001.pdf]

Supplementary Material Table S1. Serbian version of the DVISS ($DVISS_{SR}$).

<table>
<tr><th colspan="5">Upitnik o disfunkcionalnom mokrenju i inkontinenciji</th></tr>
<tr><th>Pitanja</th><th colspan="4">Odgovori</th></tr>
<tr><td>1. Da li je Vaše dete mokro tokom dana?</td><td>Ne</td><td>Ponekad</td><td>1-2 puta na dan</td><td>Uvek</td></tr>
<tr><td>2. Koliko se Vaše dete umokri tokom dana?</td><td>Vlaži donji veš</td><td>Navlaži malo pantalone</td><td colspan="2">Navlaži dosta pantalone</td></tr>
<tr><td>3. Da li je Vaše dete mokro tokom noći?</td><td>Ne</td><td>1-2 noći nedeljno</td><td>3-5 noći nedeljno</td><td>6-7 noći nedeljno</td></tr>
<tr><td>4. Koliko se Vaše dete umorki tokom noći?</td><td colspan="2">Navlaži malo posteljinu kreveta</td><td colspan="2">Navlaži dosta posteljinu kreveta</td></tr>
<tr><td>5. Koliko puta Vaše dete mokri?</td><td colspan="2">Manje od 7 puta dnevno</td><td colspan="2">7 ili više puta dnevno</td></tr>
<tr><td>6. Moje dete se napreže tokom mokrenja</td><td colspan="2">Ne</td><td colspan="2">Da</td></tr>
<tr><td>7. Moje dete oseća bol tokom mokrenja</td><td colspan="2">Ne</td><td colspan="2">Da</td></tr>
<tr><td>8. Moje dete mokri povremeno</td><td colspan="2">Ne</td><td colspan="2">Da</td></tr>
<tr><td>9. Moje dete ima potrebu da ide da mokri ubrzo po završetku prethodnog mokrenja</td><td colspan="2">Ne</td><td colspan="2">Da</td></tr>
<tr><td>10. Moje dete ima iznenadni osećaj za potrebom da odmah mokri</td><td colspan="2">Ne</td><td colspan="2">Da</td></tr>
<tr><td>11. Moje dete zadržava mokrenje tako što prekrsti noge</td><td colspan="2">Ne</td><td colspan="2">Da</td></tr>
<tr><td>12. Moje dete se umokrava na putu ka toaletu</td><td colspan="2">Ne</td><td colspan="2">Da</td></tr>
<tr><td>13. Moje dete nema pražnjenje creva svaki dan</td><td colspan="2">Ne</td><td colspan="2">Da</td></tr>
<tr><td>Pitanje o kvalitetu života: Ukoliko Vaše dete ima neki od gore navedenih simptoma, da li to utiče na njegov porodični, socijalni i školski život?</td><td>Ne</td><td>Ponekad</td><td>Da, malo utiče</td><td>Da, mnogo utiče</td></tr>
</table>
